# Effect of Al_2_O_3_ Buffer Layers on the Properties of Sputtered VO_2_ Thin Films

**DOI:** 10.1007/s40820-017-0132-x

**Published:** 2017-02-14

**Authors:** Dainan Zhang, Tianlong Wen, Ying Xiong, Donghong Qiu, Qiye Wen

**Affiliations:** 1 State Key Laboratory of Electronic Films and Integrated Devices, University of Electronic Science and Technology of China, Chengdu, 610054 People’s Republic of China; 2 Department of Electrical and Computer Engineering, University of Delaware, Newark, DE 19716 USA

**Keywords:** Al_2_O_3_, Buffer layers, Atomic layer deposition, VO_2_ thin films, Heterostructure

## Abstract

VO_2_ thin films were grown on silicon substrates using Al_2_O_3_ thin films as the buffer layers. Compared with direct deposition on silicon, VO_2_ thin films deposited on Al_2_O_3_ buffer layers experience a significant improvement in their microstructures and physical properties. By optimizing the growth conditions, the resistance of VO_2_ thin films can change by four orders of magnitude with a reduced thermal hysteresis of 4 °C at the phase transition temperature. The electrically driven phase transformation was measured in Pt/Si/Al_2_O_3_/VO_2_/Au heterostructures. The introduction of a buffer layer reduces the leakage current and Joule heating during electrically driven phase transitions. The *C*–*V* measurement result indicates that the phase transformation of VO_2_ thin films can be induced by an electrical field.

**Electronic supplementary material:**

The online version of this article (doi:10.1007/s40820-017-0132-x) contains supplementary material, which is available to authorized users.

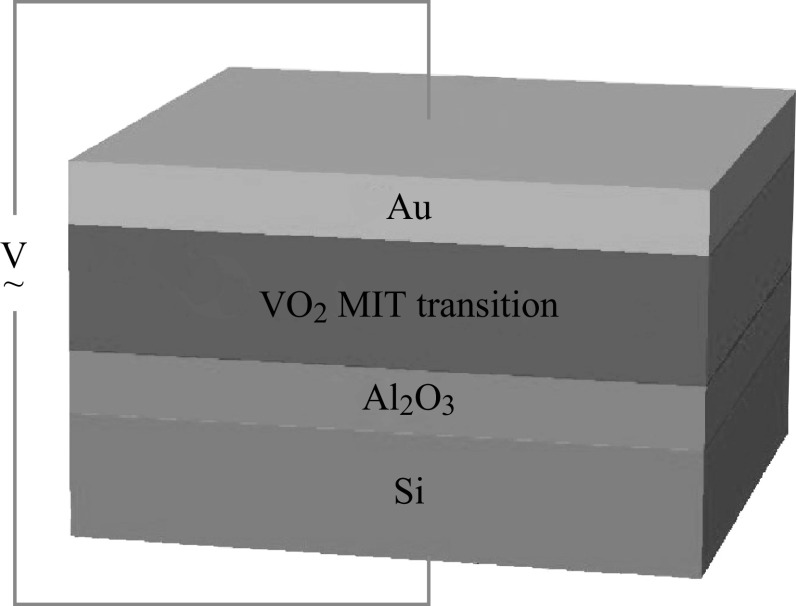

## Highlights

- High-quality VO_2_ thin films were obtained on silicon substrates by introducing an Al_2_O_3_ buffer layer prepared by atomic layer deposition (ALD) under different growth conditions.
- The fast, electrically driven phase transition of VO_2_ thin films was studied, and a possible mechanism was proposed according to the *C–V* measurement at a high frequency.

## Introduction

VO_2_ thin films have generated a considerable interest among scientists over the past decades owing to their near-room-temperature phase transformation [1]. VO_2_ thin films undergo transition from a reversible monoclinic (M phase) to a rutile (R phase) structural phase at approximately 65 °C, which is accompanied by a dramatic change in their electrical and optical properties [2–6]. The resistivity of VO_2_ thin films changes by three to five orders of magnitude during this phase transition. The metal-to-insulator (MIT) transition has been utilized in a variety of electronic devices such as electromagnetic wave modulators, switches [7–10], and holographic storage [11]. So far, the applications of VO_2_ thin films have been mainly explored for infrared and millimeter waves [10, 12]. Recently, VO_2_ thin films have been used for terahertz (THz)-wave devices [7]. In 2010, VO_2_ nanowires were deposited on glass substrates using photolithography and magnetron sputtering and used for the thermal modulation of THz waves by Wen et al. [7]. The research results showed that the transmittance of THz waves reduced by 65% after traveling through VO_2_ nanowire arrays during the MIT transition [7].

In 2013, VO_2_ thin films with silver nanowire antenna arrays were deposited on a silicon substrate [13]. A reduction in the relative refractive index and a blue shift of the resonance frequency were observed, owing to changes in the dielectric constant of VO_2_ thin films during heating [13]. Similarly, Seo et al. [8] created a rectangular hole array based THz wave antenna for controlling the transmittance of THz waves from 0.2 to 2 THz using the phase transition of VO_2_ thin films.

VO_2_ thin films have exhibited good potential for use in THz wave devices. However, compared to the electrical modulation in graphene [14–17] and the optical modulation in silicon [18–20], it is difficult to obtain a comparable response speed via the thermal modulation of THz waves using the phase transition of VO_2_ thin films. Consequently, it is important to explore the fast phase transition of VO_2_ thin films induced by an electrical field.

In many applications, the integration of VO_2_ thin films with silicon technology and processing is desired, which requires the growth of VO_2_ thin films on a silicon substrate [21]. However, there is a large lattice mismatch between the VO_2_ thin films and silicon/silicon oxide substrates. The direct deposition of VO_2_ thin films on silicon/silicon oxide often yields an inferior crystalline texture, resulting in a small change of resistivity and a large thermal hysteresis during MIT [21, 22]. Furthermore, the oxygen diffusion at the VO_2_/Si interface adversely affects the quality of the deposited VO_2_ thin films [22].

It has been proposed that using yttrium-stabilized zirconia (YSZ) as a buffer layer aids the growth of VO_2_ thin films on a silicon substrate [21]. A YSZ buffer layer of 30–145 nm can reduce the thermal hysteresis temperature to 6 K and result in a resistance change of three orders of magnitude [21]. However, owing to the instability of the microstructure of YSZ, it is necessary to study a new buffer layer for VO_2_ thin film deposition on a silicon substrate to achieve a better performance.

Previously, we used a dense Al_2_O_3_ thin film as the buffer layer to improve the quality of VO_2_ thin films grown on a silicon substrate. This layer was grown via atomic layer deposition (ALD) [5]. The introduction of the Al_2_O_3_ buffer layer significantly improved the resistivity changes and the thermal hysteresis of VO_2_ thin films during the MIT transition. An electrical-field-induced phase transformation was observed owing to the insulating Al_2_O_3_ layer.

However, it is challenging to obtain a pure phase of VO_2_ in thin films, and much knowledge about the phase transformation of VO_2_ is still unclear. Here, we have deposited VO_2_ thin films under different conditions and investigated their optical and electrical properties. It was observed that the partial pressure of oxygen gas during sputtering has a significant impact on the phase of the deposited thin film. Pure VO_2_ can be obtained on the Al_2_O_3_ buffer layer by optimizing the growth conditions. In contrast, it is difficult to obtain a pure VO_2_ phase on a naked silicon substrate. In addition, the phase transformation induced by an electrical field was also studied. The capacitance–voltage curves indicate that the phase transformation occurs through the voltage-driven nucleation and the growth of metallic regions in VO_2_ thin films. The high-quality VO_2_ thin films grown on silicon substrates have great potential for applications in many functional devices.

## Experimental Methods

In this study, p-type silicon (001) (with a resistivity *ρ* of approximately 1 Ω cm) was used as substrate, and cleaned by acetone, ethylene, and deionized water before deposition. The pretreated silicon substrate was loaded into a chamber for Al_2_O_3_ thin film deposition via plasma-enhanced atomic layer deposition (PEALD). Pure trimethylaluminum (TMA) was used as the precursor chemical. Al_2_O_3_ thin films with thicknesses ranging from 25 to 50 nm were deposited and used as the buffer layers for the growth of VO_2_ thin films. Subsequently, the Al_2_O_3_-coated silicon substrate was transferred into a magnetron sputtering machine. In addition, naked silicon substrates were loaded into the same batch for comparison. Pure 4-inch vanadium (99.99%) was used as a target, and pure oxygen and argon gases were used as the reaction and sputtering gases. The base pressure was <3 × 10^−4^ Pa. During the deposition, the substrate temperature was maintained between 450 and 550 °C with a sputtering power of 200 W. VO_2_ thin films with thicknesses ranging from 80 to 200 nm were obtained after a deposition time of 10–30 min. The chamber pressure was approximately 1 Pa. The partial pressure of oxygen varied from approximately 4–5%.

The deposited Al_2_O_3_ buffer layer and VO_2_ thin films were evaluated using scanning electron microscopy (SEM, JSM-7600F), X-ray diffraction (XRD, DX-2700), and atomic force microscopy (AFM, SPA-300HV). Two gold electrodes of 1 mm separated by a distance of 6.5 µm were deposited to measure the in-plane resistivity. The silicon substrate was coated with platinum, and a gold pad with an area of 100 × 100 µm^2^ was deposited on the VO_2_ thin films for perpendicular measurement. The electrical resistivity was measured via a standard four-point measurement method using a Keithley 2400 Source Meter. The *I*–*V* curve and the capacitance as a function of voltage and frequency were measured using an Agilent 4156C system.

## Results and Discussion

Al_2_O_3_ thin films deposited via PEALD were dense, smooth, with a clear interface, and high dielectric constant (i.e., *k* of approximately 8.8). They were thermally and chemically stable. Furthermore, the quality of the VO_2_ thin film was significantly improved by depositing it on the Al_2_O_3_ buffer layer. A 3D AFM image of the Al_2_O_3_ thin film is shown in Fig. S1. The roughness of the deposited Al_2_O_3_ films was approximately 0.2 nm, which is comparable with that of the polished silicon substrates. In our study, Al_2_O_3_ thin films with a thickness of 25 nm were used as the buffer layers for VO_2_ thin film deposition.

**Fig. 1:**
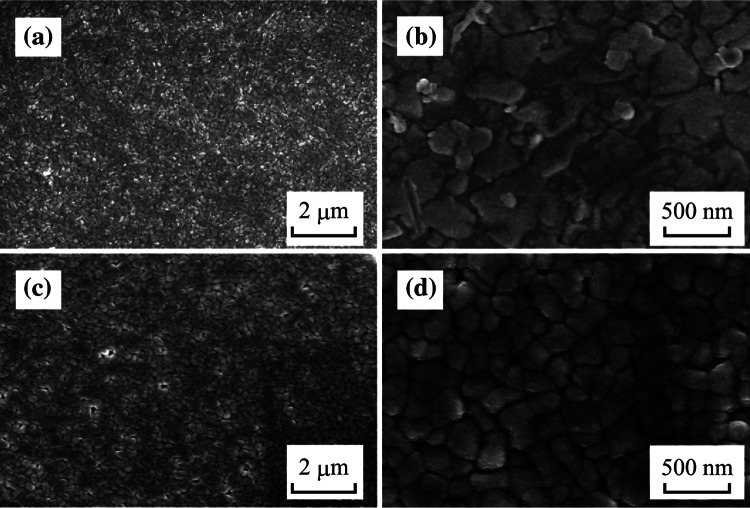
SEM images of VO_2_ thin films: **a, b** deposited on p-Si substrate, and **c**, **d** deposited on Al_2_O_3_ buffer layers. Here, **a, c** are shown in low magnification, and **b**, **d** are shown in high magnification

Figure 1 shows the SEM images of the sputtered VO_2_ thin films on a silicon substrate and on the deposited Al_2_O_3_ buffer layers at different magnifications. It was observed that both the VO_2_ thin films were polycrystalline but with different microstructures. The grains of the VO_2_ thin films grown on the silicon substrate were small, irregular, and nonuniform in size. In contrast, the grains of the VO_2_ thin films grown on the Al_2_O_3_ buffer were large, smooth, dense, and uniform in size. In the case of polycrystalline VO_2_ thin films, the phase transformation can be affected by their crystallinity, grain size, density, and grain boundaries [23]. The discontinuous phase transformation is mainly caused by the energy barriers imposed by the grain boundaries [23]. Consequently, larger and denser grains benefit the phase transformation of the VO_2_ thin films, and they were obtained in this work by introducing the Al_2_O_3_ buffer layer, as shown in Fig. 1.

Vanadium has multiple valence states (+2, +3, +4, and +5) in oxides, which is a challenge for obtaining a pure phase of VO_2_ thin films. In our experiment, we observed that there was only a small window for the oxygen partial pressure to produce pure VO_2_ thin films on the Al_2_O_3_ buffer layer. A slight difference in oxygen pressure during magnetron sputtering can significantly affect the phase of the deposited VO_x_ thin films. In this study, oxygen partial pressures of 4% and 5% were used to demonstrate their impact on the VO_2_ thin films.

**Fig. 2:**
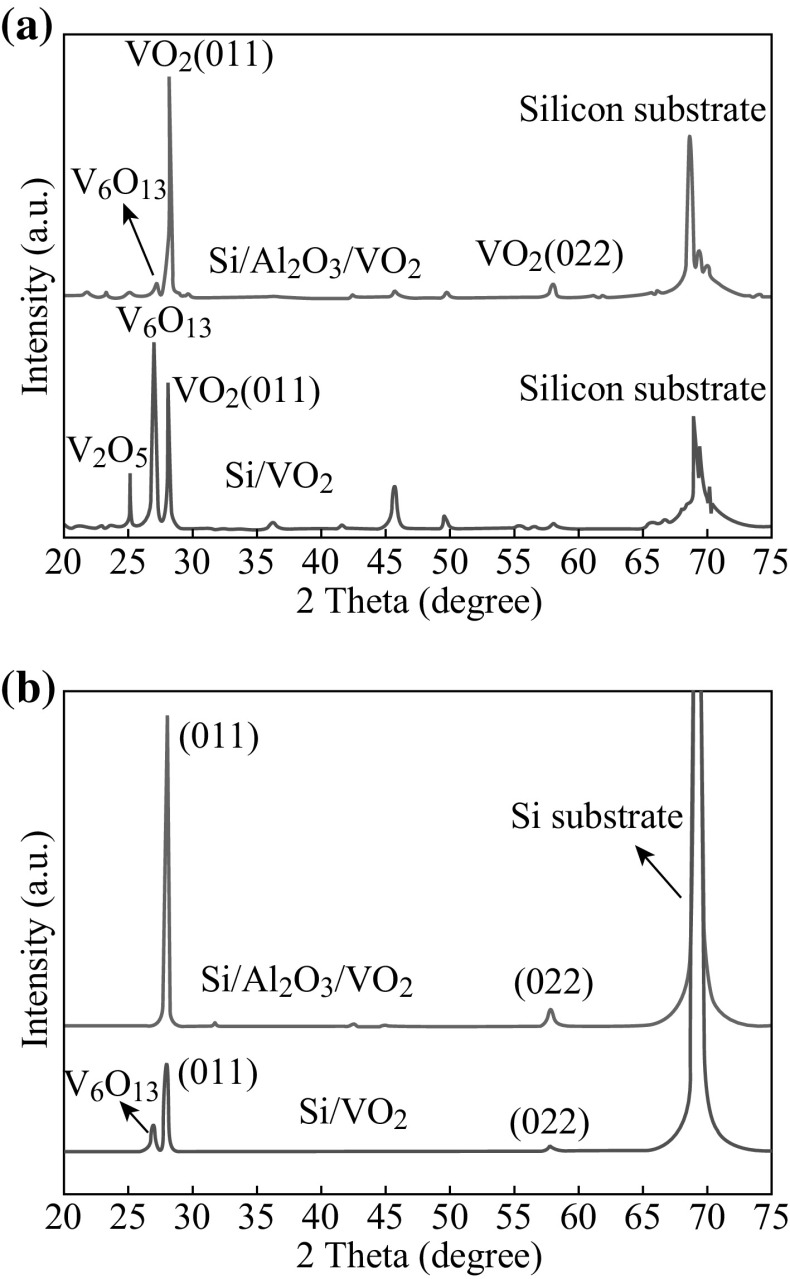
X-ray *θ*–2*θ* scan of VO_2_ thin films grown on silicon and Al_2_O_3_ buffer layers under oxygen partial pressure of 5%, and 4% in **a** and **b**, respectively

Figure 2a, b show the XRD patterns of two sets of samples prepared under oxygen pressures of 5% and 4%, respectively. In the case of oxygen partial pressure of 5%, VO_2_ thin films were obtained both on silicon and Al_2_O_3_ buffer layer. On a naked silicon substrate, V_6_O_13_ and V_2_O_5_ phases were also observed and the growth of the VO_2_ phase was significantly suppressed. In contrast, the growth of VO_2_ thin films was enhanced on the Al_2_O_3_ buffer layer, and the V_2_O_5_ phase was indiscernible by XRD for oxygen partial pressures of 5% and 4%. However, a small amount of V_6_O_13_ phase was still observed.

The undesired phases of V_6_O_13_ and V_2_O_5_ were oxygen rich as compared to the desired VO_2_ phase. Consequently, the oxygen partial pressure was reduced to 4% during the magnetron sputtering. On the naked silicon substrate, the V_2_O_5_ phase was not discernible for the reduced oxygen partial pressure. Although a small amount of V_6_O_13_ phase was still present in the deposited thin films, the growth of the VO_2_ phase was highly enhanced compared to the deposition at the oxygen partial pressure of 5%. Moreover, a pure VO_2_ phase was obtained for the thin films deposited on the Al_2_O_3_ buffer layer at the oxygen partial pressure of 4%, and the growth of the VO_2_ phase in the (011) direction was highly enhanced, which was evident from the high intensity of the (011) diffraction peak.

The enhanced VO_2_ growth on the Al_2_O_3_ buffer layer could be the result of the reduced lattice mismatches and the oxygen diffusion at the Al_2_O_3_/VO_x_ interface. The XRD patterns did not show signatures of the ALD-deposited Al_2_O_3_ thin films, indicating the amorphous nature of the buffer layers, which is consistent with previous research results [24, 25]. The amorphous Al_2_O_3_ buffer layer did not strain the lattice of the VO_2_ thin films during growth, unlike naked silicon. Therefore, the VO_2_ thin films grown on the Al_2_O_3_ buffer layers were much more homogenous than those grown on the silicon substrate, as shown in Fig. 1. Further, the Al_2_O_3_ buffer layers promoted texture growth along the (011) direction, which was also reported by other groups [26, 27].

Further, the Al_2_O_3_ buffer layer was chemically stable, preventing the interdiffusion of oxygen at the Al_2_O_3_/VO_2_ interface. Oxygen atoms can easily diffuse at the Si/VO_2_ interface at 450–550 °C, which renders the control of the stoichiometric composition of the VO_2_ thin films difficult. Therefore, by introducing the Al_2_O_3_ buffer layer on a silicon substrate, the microstructure and stoichiometric composition were easier to control, resulting in higher quality VO_2_ thin films. Notably, pure VO_2_ thin films can also be deposited by magnetron sputtering from a vanadium target by carefully controlling the growth conditions [27]. However, the use of the Al_2_O_3_ buffer layer renders the deposition window wider and the electrical properties stronger, which will be discussed later.

The thermally induced phase transformations were investigated under different deposition conditions and microstructures. The sheet resistance of the thin films was measured as a function of temperature. The results are shown in Fig. S2 (oxygen partial pressures of 5%) and Fig. S3 (oxygen partial pressures of 4%). Table 1 lists the sheet resistances of the samples at 25 and 85 °C, and the transition temperatures during heating and cooling, which are calculated from Fig. S2 and Fig. S3. The resistivity ratio of the thin films before (*T* = 25 °C) and after (*T* = 25 °C) phase transition were significantly improved when deposited on the Al_2_O_3_ buffer layers over a naked silicon substrate, owing to the high resistivity of the thin films at room temperature (25 °C).

**Table 1:** Sheet resistance and phase transformation temperature

Sample	PO_2_ (%)	*ρ*_25 °C_ (kΩ/sq)	*ρ*_85 °C_ (kΩ/sq)	*ρ*_25 °C_/*ρ*_85 °C_	*T*_Cool_ (°C)	*T*_Heat_ (°C)	Δ*T* (°C)
Si/VO_2_	5	4.5	0.025	180	56	65	9
	4	23	0.034	676	51.5	59.5	8
Si/AI_2_O_3_/VO_2_	5	169	0.07	2414	57	63	6
	4	106	0.013	8153	56	60	4

As shown in Table 1, the sheet resistance of the thin films deposited on the naked silicon was much smaller than that on the Al_2_O_3_ buffer layer, which could be the result of the presence of the V_6_O_13_ phase. The V_6_O_13_ phase undergoes an MIT transition at −123 °C, and it is in a metallic state at room temperature [28]. In Fig. 2, the thin films possessed a significant amount of V_6_O_13_ phase when deposited on the naked silicon, whereas the growth of V_6_O_13_ was suppressed when deposited on the Al_2_O_3_ buffer layer. The resistivity changes in the VO_2_ thin films were further increased during the phase transition when deposited at the oxygen partial pressure of 4%. In addition, the temperature of thermal hysteresis was reduced from 9 °C on the naked silicon to 4 °C on the Al_2_O_3_ buffer layer at the oxygen partial pressure of 4%.

**Fig. 3:**
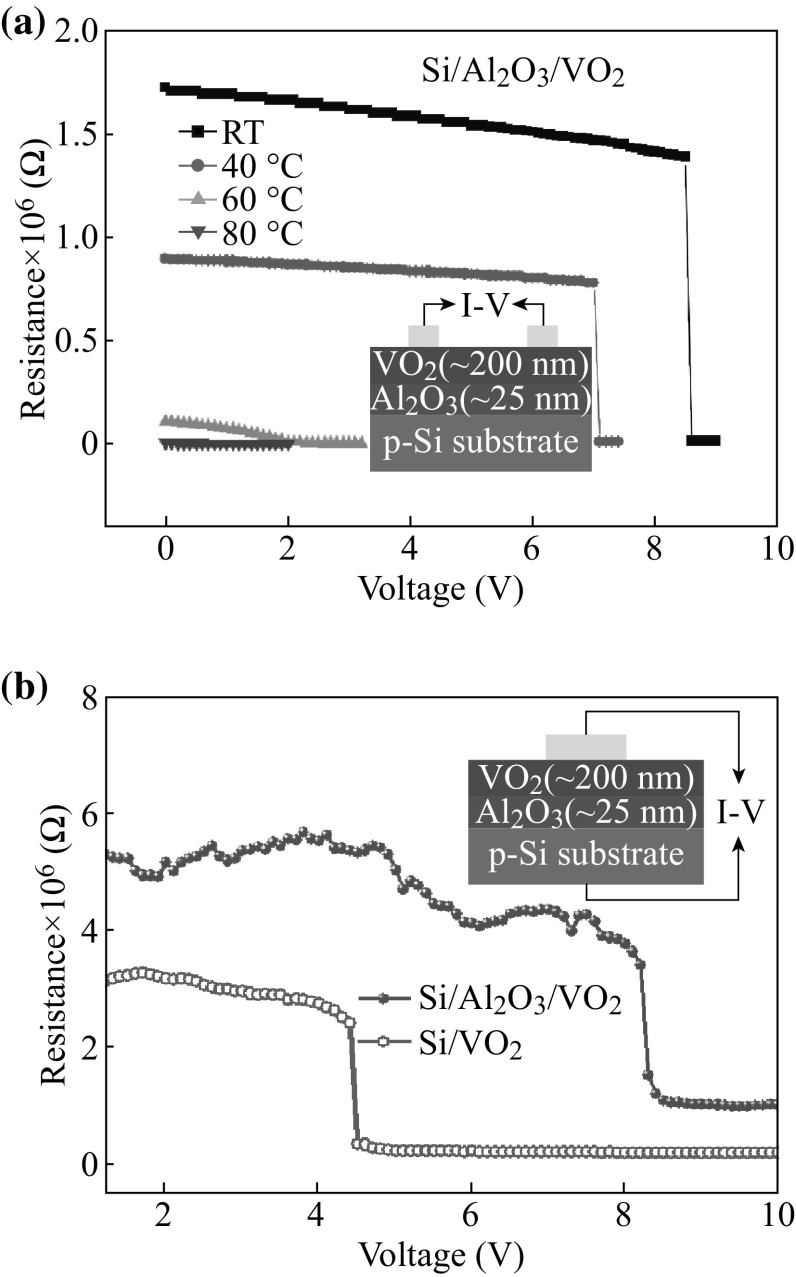
**a** Resistance of VO_2_ thin films on Al_2_O_3_ buffer layers measured along the in-plane direction, and **b** the resistance measured along the perpendicular direction for VO_2_ thin films deposited on Al_2_O_3_ buffer layers and naked silicon substrate

Electrodes were deposited on Si/Al_2_O_3_/VO_2_ to investigate the electrically driven phase transformation. The resistance of the VO_2_ thin films was measured along the in-plane and perpendicular directions, as shown in the insets of Fig. 3a, b, respectively. Two gold electrodes of 1 mm, separated by 6.5 µm, were deposited via thermal evaporation for the in-plane measurement. The Al_2_O_3_ buffer layers could prevent current leakage through the p-Si. At room temperature, the resistance had a magnitude of 10^6^ Ω when no bias was applied to the electrodes. As the voltage bias was increased from 0 V, initially the resistance decreased gradually. However, when the voltage approached 8.5 V, an abrupt decrease of resistance was observed at room temperature, indicating a phase transformation.

Furthermore, an electrically driven phase transition was observed as the environmental temperature was increased to 40 °C, but the thermally induced phase transformation still did not occur. However, the resistivity was small at 40 °C than that at room temperature. Further, the switching voltage was reduced to approximately 7 V, which was lower than the value at room temperature. Therefore, thermal energy can assist the electrically driven phase transformation of VO_2_ thin films. When the environmental temperature approached the value of the thermal phase transformation temperature at 60 °C, the resistance change during the electrically driven phase transition was very small, as shown by the green curves in Fig. 3a. At 80 °C, the VO_2_ thin films were in metallic states, and no abrupt change in resistance was observed. Notably, the switching voltage monotonically decreased as the temperature increased.

The electrically driven phase transformation could be a result of the applied electrical field or Joule heating. In this regard, we are prone to believe that the phase transformation was induced by the applied electrical field. For verification, we measured the resistance of the VO_2_ thin films along the perpendicular direction, as shown in the inset of Fig. 3b. The insulating Al_2_O_3_ buffer layer could effectively decrease the electrical current passing through the VO_2_ thin films. The resistance was measured as a function of the applied voltage, as shown in Fig. 3b. The phase transformation occurred at 8.4 V for the Pt/Si/Al_2_O_3_/VO_2_/Au stack, and at 4.4 V for the Pt/Si/VO_2_/Au stack.

In Fig. 3a, the resistance of VO_2_ is negligible after the phase transformation. Therefore, we estimated that the apparent resistances of the silicon substrate, Al_2_O_3_ buffer layer, and VO_2_ thin films immediately before the phase transformation were 2 × 10^5^, 8 × 10^5^, and 3 × 10^6^ Ω, respectively. A switching electrical field of 3 × 10^7^ V m^−1^ for the Pt/Si/Al_2_O_3_/VO_2_ stack was estimated. When the VO_2_ thin films were deposited on the silicon substrate, their resistance was estimated to be 2.45 × 10^6^ Ω before the phase transformation, and the switching electrical field was 2 × 10^7^ V m^−1^. The decrease in the switching field of the Pt/Si/VO_2_/Au stack could be a result of the microstructure of the deposited thin films.

**Fig. 4:**
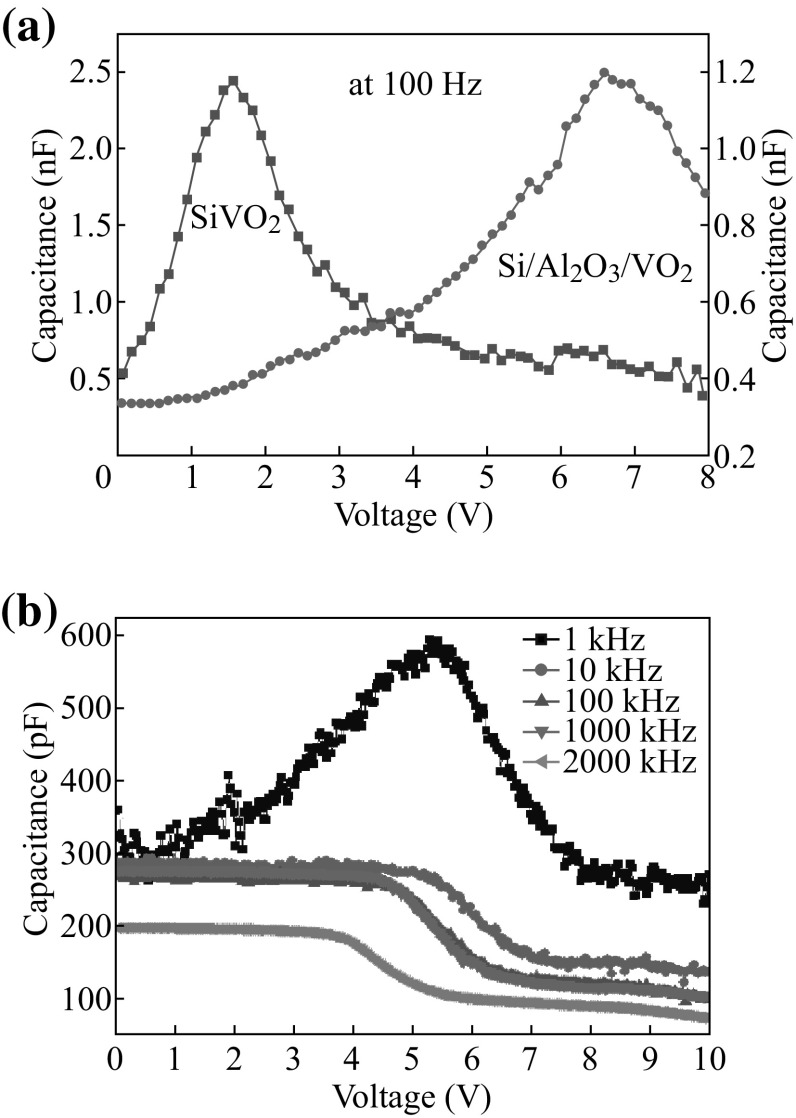
**a** Capacitance of Pt/Si/Al_2_O_3_/VO_2_/Au and Pt/Si/VO_2_/Au stacks as a function of voltage at 100 Hz, and **b** capacitance of Pt/Si/Al_2_O_3_/VO_2_/Au stacks as a function of voltage at different frequencies

In order to further investigate the electrically driven phase transformation of the VO_2_ thin films, the capacitances of the Pt/Si/Al_2_O_3_/VO_2_/Au and Pt/Si/VO_2_/Au stacks were measured under an alternating field at 100 Hz. The result is shown in Fig. 4a. As the voltage increased, the capacitance of both the stacks increased and subsequently decreased, with a maximum value observed at 1.5 V for Pt/Si/VO_2_/Au and at 6.8 V for Pt/Si/Al_2_O_3_/VO_2_/Au. The capacitance–voltage curves were very different from those of typical MOS capacitors. We attribute this unusual behavior to the electrically driven phase transformation of the VO_2_ thin films. As the voltage increased, some regions of the VO_2_ thin films were transformed from insulating to metallic.

**Fig. 5:**
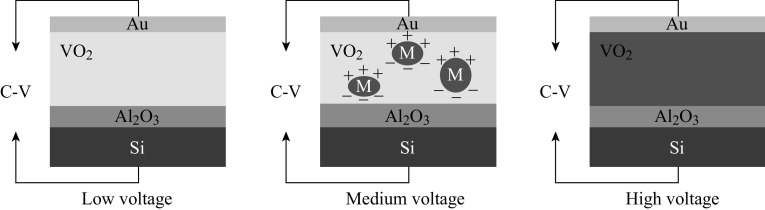
Schematic mechanism of electrically driven phase transformation of VO_2_ thin films

Figure 5 shows the charge carriers were delocalized in the metallic region and oscillated with the AC field. It resulted in an increase in the dielectric constant of the VO_2_ thin films. As the metallic region was percolated at high voltages, the capacitance decreased. The maximum capacitance during the phase transformation, which were triggered by temperature [29], was also observed in other MIT transitions. As shown in Fig. 4a, the nucleation and growth of the conducting regions can be induced gradually via a fast charge carrier injection during the AC oscillation [30]. These regions became conductive as percolation was achieved.

The capacitance of the Pt/Si/Al_2_O_3_/VO_2_/Au stack was also measured as a function of voltage at different frequencies, as shown in Fig. 4b. At 1 kHz, the maximum capacitance was observed at a lower voltage of 5.2 V compared to the corresponding voltage value observed at 100 Hz. As the frequency of the AC field increased to 10 kHz, the maximum capacitance disappeared as the voltage increased, indicating that the oscillation of the charge carriers in the metallic regions could not match the AC field. The electrically driven phase transition of the VO_2_ thin films can occur within 10 µs [31, 32]. The longer response time in our experiment could be a result of the large time constant RC owing to the presence of the Al_2_O_3_ layer. However, the phase transformation, indicated by the drop in capacitance, shifted to lower voltages as the frequency increased. This could be the result of easier nucleation of the new phase at high frequencies.

## Conclusions

Al_2_O_3_ thin films were used as buffer layers to improve the growth of VO_2_ thin films on silicon substrate. Pure VO_2_ phases were obtained by depositing on the Al_2_O_3_ buffer layer at an oxygen partial pressure of 4%. Accordingly, a large resistance change and a small hysteresis were obtained for the thermally induced phase transformation. Furthermore, the electrical-field-induced phase transformation of VO_2_ thin films was also investigated. By measuring the capacitance at different voltages and frequencies, we propose that the phase transformation was initialized by the formation of metallic regions in the insulating VO_2_ matrix. Our work provides a facile method to deposit VO_2_ thin film on silicon substrate, which paves the way for fabricating VO_2_-based devices such as fast electrical modulation of THz waves.

## Electronic supplementary material

Below is the link to the electronic supplementary material.

Supplementary material 1 (PDF 527 kb)
