# Supplementary material for: Effect of Al2O3 Buffer Layers on the Properties of Sputtered VO2 Thin Films
Source: Nanomicro Lett. 2017 Feb 14;9(3):29. doi: 10.1007/s40820-017-0132-x (PMC6199022; doi:10.1007/s40820-017-0132-x)
Supplement: Supplementary file 1 — Supplementary material 1 (PDF 527 kb) [file 40820_2017_132_MOESM1_ESM.pdf]

Supplemental Information for

Effect of Al_2O_3 Buffer Layers on the Properties of Sputtered VO_2 Thin Films

Dainan Zhang[1,2], Tianlong Wen[1,*], Ying Xiong[1], Donghong Qiu[1], Qiye Wen[1]

[1]State Key Laboratory of Electronic Films and Integrated Devices, University of Electronic Science and Technology of China, Chengdu 610054, People's Republic of China

[2]Department of Electrical and Computer Engineering, University of Delaware, Newark, Delaware, 19716, USA

*Corresponding author. E-mail: halong@uestc.edu.cn

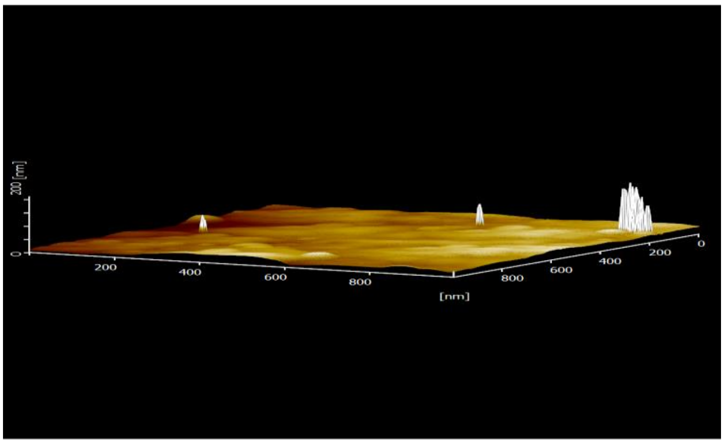

Figure S1 The AFM image of Al_2O_3 thin film

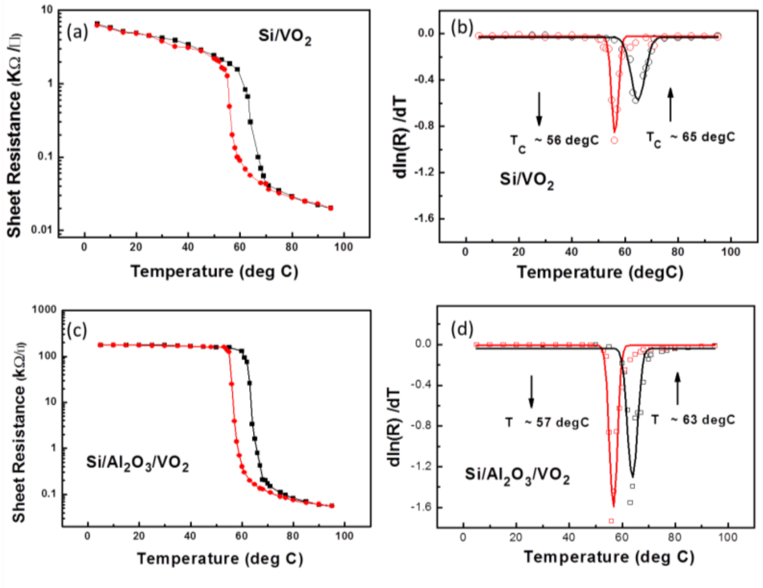

Figure S2 Sheet resistance of **a** Si/VO_2 and **c** Si/Al_2O_3/VO_2 grown under 5% oxygen partial pressure as a function of temperature, **b** and **d** shows the derivatives of the curves in **a** and **c** respectively.

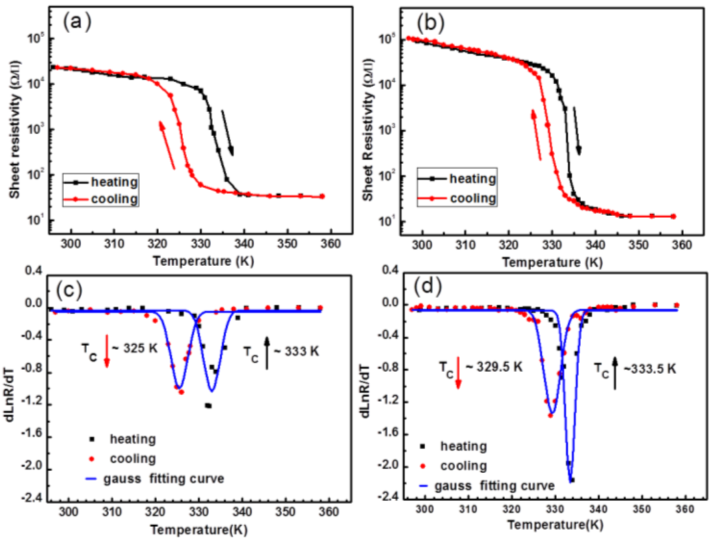

Figure S3 Sheet resistance of **a** Si/VO_2 and **b** Si/Al_2O_3/VO_2 grown under 4% oxygen partial pressure as a function of temperature, **c** and **d** shows the derivatives of the curves in **a** and **c** respectively.
